# Primary care providers' knowledge, attitudes, and practices related to prediabetes in China: A cross-sectional study

**DOI:** 10.3389/fpubh.2023.1086147

**Published:** 2023-02-23

**Authors:** Linhua Pi, Jianru Yan, Dongxue Fei, Ying Zheng, Xiajie Shi, Zhen Wang, Zhiguang Zhou

**Affiliations:** ^1^Department of Metabolism and Endocrinology, The Second Xiangya Hospital, Central South University, Changsha, China; ^2^Key Laboratory of Diabetes Immunology (Central South University), Ministry of Education, Changsha, China; ^3^National Clinical Research Center for Metabolic Diseases, Changsha, China; ^4^Department of Metabolic Endocrinology, The First People's Hospital of Pingjiang, Yueyang, China; ^5^Clinical Nursing Teaching and Research Section, The Second Xiangya Hospital of Central South University, Changsha, China; ^6^Center for Medical Research, The Second Xiangya Hospital, Central South University, Changsha, China

**Keywords:** prediabetes, KAP, primary care providers, China, cross-sectional study

## Abstract

**Background:**

The management of prediabetes has great clinical significance, and primary care providers (PCPs) play important roles in the management and prevention of diabetes in China. Nevertheless, little is known about PCPs' knowledge, attitudes, and practices (KAP) regarding prediabetes. This cross-sectional study aimed to assess the KAP regarding prediabetes among PCPs in the Central China region.

**Methods:**

This cross-sectional study was conducted using self-administered KAP questionnaires among PCPs from Central China region.

**Results:**

In total, 720 PCPs completed the survey. Most physicians (85.8%) claimed to be aware of the adverse effects of prediabetes and reported positive attitudes toward prediabetes prevention, but the PCPs' knowledge of prediabetes and management practices showed substantial gaps. The prediabetes knowledge level and practice subscale scores of the PCPs were only 54.7% and 32.6%, respectively, of the corresponding optimal scores. Female PCPs showed higher prediabetes knowledge level scores (*p* = 0.04) and better practice scores (*p* = 0.038). Knowledge and attitude scores were inversely correlated with participants' age and duration of practice (*p* < 0.001). The PCPs who served in township hospitals had significantly higher knowledge and attitude scores than those who served in village clinics (*p* < 0.001). Furthermore, knowledge and practice scores increased with increasing professional titles. Recent continuing medical education (CME) attendance had a significant positive influence on knowledge of prediabetes (*p* = 0.029), but more than four-fifths of the surveyed PCPs did not participate in diabetes-related CME in the past year.

**Conclusions:**

Substantial gaps were observed in PCPs' knowledge and practices regarding prediabetes in the Central China region. CME programmes were under-utilized by PCPs. Structured programmes are required to improve PCPs' prediabetes-related knowledge and practices in China.

## Introduction

Prediabetes is a borderline glycaemic status presenting with impaired glucose metabolism that does not meet the diagnostic criteria for diabetes. According to the recent guidelines from the American Diabetes Association (ADA), prediabetes is defined by fasting plasma glucose (PG) level of 5.6–6.9 mmol/l, a 2-h PG level of 7.8–11.0 mmol/l in a 75-g oral glucose tolerance test (OGTT), and a hemoglobin A1c level of 5.7%−6.4% (1). A recent national survey indicated that the prevalence of prediabetes was 35.2% among adults living in China; thus, approximately 357 million Chinese have prediabetes (2). Furthermore, prediabetes is associated with a high risk of progression to overt type 2 diabetes (3) and may confer an increased risk of premature mortality (4, 5) and multiple chronic complications observed in established diabetes (6, 7). Numerous studies have confirmed that lifestyle interventions and medications are effective in delaying or preventing the development of diabetes (8–10).

Despite the success of preventive interventions, nearly 90% of individuals with prediabetes are not informed of their conditions by health providers (11) and most American patients with prediabetes do not receive appropriate interventions (12, 13). The situation in China may be worse, especially in the rural regions. Primary care physicians (PCPs) from townships or village doctors play a vital role in diabetes prevention by screening for and managing prediabetes. Thus, PCPs' knowledge of prediabetes and their attitudes and practices related to this condition are essential elements of prediabetes management. Previous studies conducted in the US (14, 15) and Latin America (16) indicated significant gaps in the knowledge of prediabetes among PCPs as well as inadequate detection and treatment of this condition by PCPs. Thus, understanding PCPs' knowledge, attitudes, and practices (KAP) related to prediabetes can facilitate the development of tailored strategies to improve knowledge, change attitudes, and address poor practices. To the best of our knowledge, PCPs' KAP related to prediabetes in China has not been described to date. Therefore, this study aimed to assess PCPs' knowledge of prediabetes as well as their attitudes toward prediabetes and prediabetes care practices in the Central China region. We also aimed to evaluate the association between provider characteristics and prediabetes-related KAP.

## Methods

This cross-sectional study was conducted between May and August 2022. Letters of invitation were sent to PCPs from township hospitals or village doctors across Yueyang, Huaihua, and Yongzhou cities in Hunan Province, Central South China. Those who agreed to participate were provided with a questionnaire through an online platform in China, which provided functions equivalent to Amazon Mechanical Turk. This study was approved by the Ethics Committee of the Second Xiangya Hospital of Central South University, and written consent was obtained from each participant.

The self-administered questionnaire included multiple-choice and open-ended questions evaluating participants' prediabetes KAP and was developed in line with the concepts proposed by a Johns Hopkins University group (15). A pre-test was conducted among 10 PCPs to test the reliability and improve the clarity and interpretability of the questionnaire. The questionnaire consisted of two sections. The first section focused on PCPs' sociodemographic characteristics (sex and age), practice setting (township hospital or village clinic), physician seniority level, time since graduation in general medicine, history of diabetes in a first-degree relative, and experience of continuing medical education (CME) programmes related to diabetes in the past year. The second section was developed according to the latest ADA criteria and the Chinese Diabetes Society guidelines released in 2020 (17). It consisted of 16 questions on the participants' prediabetes-related knowledge (6 questions), attitudes (6 questions), and practices (4 questions). Questions related to knowledge and practice were evaluated using a two-point scale (1 = correct, 0 = false, and not sure). The questions related to attitude used a five-point Likert scale (1 = positive attitude, 0 = negative practice, or uncertain). Additionally, we asked providers to select what they considered significant challenges to lifestyle modification from a list of potential barriers drawn from prior studies regarding similar topics (14, 16) and asked them to list other possible potential barriers they encountered in daily clinical practice.

### Statistical analyses

Data were extracted from the questionnaires and analyzed using SPSS version 25.0 (IBM Corporation, Chicago, IL, USA). Descriptive data were presented as numbers, percentages, means, and standard deviations, depending on whether the variables were categorical or continuous. The association between PCPs' characteristics and respondents' KAP was evaluated by Chi-square tests and *t*-tests, as appropriate. Multiple linear regression models were used to examine the association between the predictor variables and the KAP scores. Statistical significance was set at *P* < 0.05.

## Results

### Sociodemographic and other characteristics of the participants

This study enrolled 720 PCPs from the three cities in Hunan Province, Central China. The characteristics of the study participants are summarized in Table 1. The mean age of the participants was 44.7 ± 9.8 years. Among the participants, 65.5% were male, and 260 and 460 physicians worked in township hospitals and village clinics, respectively. The physicians involved in this study included 630 resident, 79 attending, and 11 senior physicians. Among them, 117 had practiced for < 10 years, 124 for 10–20 years, and 479 for >20 years. The majority (73.1%) of respondents had not participated in CME programmes related to diabetes in the past year, and 113 respondents had a family history of diabetes.

**Table 1:** Characteristics of the study participants (*N* = 720).

**Provider characteristics**	**Mean ±SD**	**Number (*n*)**	**Percentage (%)**
**Sex**			
Male		472	65.5%
Female		248	34.5%
Age (years)	44.7 ± 9.8		
≤ 40		218	30.3%
>40		502	69.7%
**Practice setting**			
Township hospital		260	36.1%
Village clinic		460	63.9%
**Professional titles**			
Resident physicians		630	87.5%
Attending physicians		79	11.0%
Senior physicians		11	1.5%
Duration of practice (years)	22.7 ± 11.7		
< 10 years		117	16.3%
10–20 years		124	17.2%
≥20 years		479	66.5%
**CME attendance during the past year**			
No		526	73.1%
Yes		194	26.9%
**Positive family history of diabetes**			
Yes		113	15.7%
No		607	84.3%

SD, standard deviation; CME, continuing medical education.

### Physicians' knowledge regarding prediabetes

The participants were asked if they knew what prediabetes was and to identify the risk factors for diabetes. While 86.5% had adequate knowledge of the definition of prediabetes, 71.9% had sufficient knowledge of the risk factors for diabetes. However, the PCPs generally lacked knowledge of glycaemic cut-offs for the diagnosis of prediabetes, and only 50.4%, 56.4%, and 26.1% could identify the correct glycaemic cut-offs for diagnosing prediabetes based on the fasting glucose level, 2-h PG level during OGTT, and hemoglobin A1c level, respectively. Overall, only 62 of the 720 PCPs (8.6%) correctly identified all three glycaemic cut-off values for the diagnosis of prediabetes, while 116 PCPs (16.1%) were unaware of all three cut-off values. Less than half of the PCPs (37.5%) chose 5%−7% as the recommended minimum amount of weight loss (Table 2).

**Table 2:** Knowledge of PCPs regarding prediabetes.

**Item**	**Correct choice**	**Score [0–1]**
	***n* (%)**	**Mean ±SD**
Is prediabetes an intermediate stage between normal glycemia and diabetes?	623 (86.5%)	0.87 ± 0.34
Which one is not a risk factor in prediabetes screening?	518 (71.9%)	0.72 ± 0.45
Which one is the prediabetes laboratory criteria for fasting glucose level?	363 (50.4%)	0.50 ± 0.50
Which one is the prediabetes laboratory criteria for the 2-h PG level during OGTT?	406 (56.4%)	0.56 ± 0.50
Which one is the prediabetes laboratory criteria for HbA1c level	188 (26.1%)	0.26 ± 0.44
Which one is the correct body weight loss recommendation for individuals with prediabetes?	270 (37.5%)	0.37 ± 0.48
Total	2,361 (54.7%)	3.29 ± 1.19

2-h PG, 2-h plasma glucose; OGTT, oral glucose tolerance test; HbA1c, hemoglobin A1c.

### Physicians' attitudes toward prediabetes

Regarding attitudes toward prediabetes, more than 90% of the respondents deemed that prediabetes increased the risk of diabetes development and premature mortality, and more than 80% reported that most participants with prediabetes were unaware of their condition. Meanwhile, the majority of participants showed a positive attitude toward prediabetes prevention; 78.9% thought prediabetes was reversible through regular exercise and drugs (metformin) (Table 3).

**Table 3:** Attitudes of PCPs regarding prediabetes.

**Item**	**Positive attitude**	**Score [0–1]**
	***n* (%)**	**Mean ±SD**
Prediabetes is associated with a high risk of progression to overt type 2 diabetes	662 (91.9%)	0.92 ± 0.27
Prediabetes is associated with an increased risk of premature mortality	654 (90.8%)	0.91 ± 0.28
Most individuals with prediabetes have not been diagnosed	603 (83.8%)	0.84 ± 0.37
Prediabetes is reversible	568 (78.9%)	0.79 ± 0.41
Regular exercise helps delay or prevent the transition from prediabetes to diabetes	685 (95.1%)	0.95 ± 0.22
Metformin helps delay or prevent the transition from prediabetes to diabetes	533 (74.0%)	0.74 ± 0.44
Total	3,705 (85.8%)	5.15 ± 1.12

### Physicians' prediabetes-related practices

The overall practice score was low. Diet changes and physical activity were the most frequently employed methods for prediabetes management (90.1%), followed by repeat laboratory work and follow-up clinic visits for individuals with prediabetes. Fifty (6.9%) and 177 (24.6%) of the surveyed PCPs identified the right choices recommended by the ADA guidelines and Chinese expert consensus, respectively. Only 59 (8.2%) PCPs prescribed drugs (metformin, acarbose, etc.) to patients who failed to respond to lifestyle modification (Table 4).

**Table 4:** PCPs' prediabetes-related practices.

**Item**	**Correct choice**	**Score [0–1]**
	***n* (%)**	**Mean ±SD**
Which one is your initial suggestion for individuals with prediabetes?	653 (90.1%)	0.91 ± 0.29
How long do you recommend individuals with prediabetes to repeat laboratory work?	50 (6.9%)	0.07 ± 0.25
How long do you recommend individuals with prediabetes to return for follow-up clinic visit?	177 (24.6%)	0.25 ± 0.43
Management of a patient who failure to respond to lifestyle modification	59 (8.2%)	0.08 ± 0.27
Total	939 (32.6%)	1.30 ± 0.66

### Barriers to lifestyle modification

At the time of the survey, most PCPs selected lack of recognition of the harm posed by prediabetes (94.0%), lack of diet and exercise guidance (91.4%), uncertainty of the effectiveness of lifestyle modifications (88.5%), and lack of motivation (82.2%) as barriers to lifestyle modification. Additionally, 237 (23.9%) PCPs listed a lack of resources or financial limitations as a potential barrier in the open-ended question.

### Correlations among KAP scores

The total mean scores for knowledge, attitude, and practice were 3.29, 5.15, and 1.30, respectively, out of possible scores of 6, 6, and 4, respectively. The knowledge and attitude scores showed a weak positive correlation (*p* = 0.001, *r* = 0.127), similar to the knowledge and practice scores (*p* = 0.004, *r* = 0.1107).

### Factors associated with the overall KAP scores

The associations between the KAP scores and provider characteristics are presented in Table 5. Female PCPs reported significantly more prediabetes-related knowledge and practices than males. They also reported high mean attitude scores; however, the differences were not statistically significant. The knowledge and attitude scores varied significantly according to age; the lower age group showed higher scores, but with the practice score, the trend was reversed even though the difference was not statistically significant. Working in township hospitals was a significant predictor of higher knowledge and attitude scores.

**Table 5:** Overall knowledge, attitude, and practice mean scores and provider characteristics.

**Provider characteristics**		**Knowledge score**		**Attitude score**		**Practice score**	

	***n***	**Mean (SD)**	***p***	**Mean (SD)**	***p***	**Mean (SD)**	***p***
**Sex**							
Male	472	3.20 ± 1.16	0.04	5.10 ± 1.15	0.477	1.27 ± 0.65	0.038
Female	248	3.47 ± 1.16		5.16 ± 1.10		1.38 ± 0.69	
**Age (years)**							
≤ 40	218	3.57 ± 1.16	< 0.001	5.40 ± 1.10	< 0.001	0.65 ± 0.44	0.288
>40	502	3.17 ± 1.18		5.03 ± 1.11		0.67 ± 0.30	
**Practice setting**							
Township hospital	260	3.55 ± 1.23	< 0.001	5.45 ± 1.03	< 0.001	1.35 ± 0.67	0.202
Village clinic	460	3.14 ± 1.14		5.0 ± 1.13		1.28 ± 0.66	
**Professional titles**							
Resident physicians	630	3.23 ± 1.16	0.001	5.12 ± 3.21	0.473	1.31 ± 0.68	0.041
Attending physicians	79	3.72 ± 1.31		5.26 ± 0.97		1.20 ± 0.49	
Senior physicians	11	3.82 ± 1.08		5.36 ± 0.67		1.73 ± 0.90	
**Duration of practice (years)**							
< 10 years	117	3.63 ± 1.12	< 0.001	5.33 ± 1.14	< 0.001	1.36 ± 0.65	0.265
10–20 years	124	3.43 ± 1.20		5.42 ± 0.92		1.36 ± 0.65	
≥20 years	479	3.17 ± 1.18		5.02 ± 1.15		1.28 ± 0.67	
**CME attendance**							
No	526	3.23 ± 1.19	0.029	5.22 ± 1.09	0.274	1.29 ± 0.65	0.448
Yes	194	3.45 ± 1.17		5.12 ± 1.13		1.34 ± 0.71	
**Positive family history of diabetes**							
Yes	113	3.48 ± 1.20	0.066	5.16 ± 1.13	0.339	1.40 ± 0.66	0.101
No	607	3.25 ± 1.18		5.05 ± 1.06		1.29 ± 0.66	

CME, continuing medical education.

Professional titles also showed statistically significant relationships with knowledge scores. The scores increased with increasing professional titles. The duration of practice was significantly associated with the knowledge and attitudes toward prediabetes. Respondents in the group with < 10 years of experience showed the highest knowledge score, whereas respondents in the group with 10–20 years of experience showed the highest attitude score. CME attendance during the past year had a significant positive influence on knowledge of prediabetes.

The joint effect of provider characteristics on KAP scores was evaluated using multiple regression analysis. Professional title was a significant provider characteristic that predicted the knowledge level of participants. Significant provider characteristics that predicted the attitude of participants were sex and the practice setting. However, none of the factors included in our study could predict the practice scores of the participants.

## Discussion

To improve the management of diabetes and other chronic diseases, the Chinese government implemented the National Basic Public Health Services (BPHS) programme in 2009 (18). PCPs play a crucial role in this programme. The BPHS programme offers blood glucose tests, blood pressure measurements, lifestyle consultations, and medical instructions for people with diabetes. To provide a practical tool for doctors, especially for PCPs and general practitioners, The Chinese Diabetes Society released an expert consensus regarding prediabetes in 2020 (17). However, little is known about the competence of PCPs to perform this role. We conducted the first KAP study on prediabetes among Chinese PCPs from the Central China region. In the present study, most physicians (85.8%) claimed to be aware of the harmful effects of prediabetes and reported positive attitudes toward prediabetes prevention, but a substantial gap was noted in PCPs' knowledge of prediabetes and management practices. The PCPs' knowledge level for prediabetes was 54.7% of the optimal level, while their practice subscale score was 32.6% of the optimum score. Our findings showed that female PCPs had higher levels of prediabetes knowledge and better practices. The knowledge and attitude scores were inversely correlated with the participants' age and duration of practice. PCPs who served in township hospitals had significantly higher knowledge and attitude scores than those who served in village clinics. Furthermore, knowledge and practice scores increased with increasing professional titles. We also found that CME attendance in the past year had a significant positively influence on knowledge of prediabetes.

Our study revealed a suboptimal level of prediabetes knowledge regarding diagnostic criteria among PCPs, which may lead to low rates of identification of patients with prediabetes. Only 62 of the 720 PCPs (8.6%) correctly identified all three glycaemic cut-off values for the diagnosis of prediabetes, and 16.1% were not aware of any of the glycaemic cut-off values for prediabetes diagnosis. Our findings were comparable with those obtained by other researchers in America (14, 15) and Latin America (16) who also reported large gaps in prediabetes knowledge among PCPs. While ~90% of individuals with prediabetes in the US are not informed of their conditions by healthcare providers (11), the situation in China is expected to be much worse. The low awareness of prediabetes could be partly attributed to the lack of optimal knowledge of prediabetes among PCPs. We also found that PCPs lacked knowledge of evidence-based recommendations for prediabetes. Weight loss is the most important factor in reducing the risk of incident diabetes, and evidence suggests that 5%−7% weight loss is sufficient to achieve this goal (19–21). Knowledge of this weight-loss target is essential for providing lifestyle consultations for patients with prediabetes. Previous studies have demonstrated that most patients with prediabetes do not receive appropriate interventions (12, 13). In our study, only 37.5% of respondents correctly identified the guideline recommendations for weight loss; addressing this knowledge gap may improve the management of prediabetes. We also observed that PCPs with a younger age and a shorter duration of practice possessed a higher level of knowledge than their counterparts. A previous study also showed that the diabetes knowledge of Iranian internists was inversely correlated with the participants' age and duration of practice (22). This could be attributed to the fact that younger PCPs are more likely to keep abreast with the current trends in prediabetes. An important finding of our study was that participation in CME regarding diabetes could significantly improve PCPs' prediabetes knowledge, which is in accordance with previous studies regarding diabetes knowledge among PCPs (23–25). However, more than four-fifths of the surveyed PCPs had not participated in CME regarding diabetes in the past year. Although many CME programs are available for PCPs, they are under-utilized. One possible reason might be the COVID pandemic preventing the in-person participation. As a newly emerging technology, telemedicine has demonstrated enormous potential to provide an effective intervention in health care (26, 27). To this end, telemedicine may be a feasible approach for overcome part of barriers during the COVID-19 pandemic period.

The current study indicated that most of the respondents deemed that prediabetes increased the risk of development of diabetes and premature mortality, and more than 80% believed that most patients with prediabetes were unaware of their condition. Meanwhile, the majority had a positive attitude toward prediabetes prevention: 78.9% thought prediabetes was reversible through regular exercise and drugs (metformin). Similar to the findings for the knowledge subscale, the overall attitude scores were inversely correlated with the participants' age and duration of practice, which could also be due to the fact that younger PCPs are more likely to keep abreast with current trends in prediabetes. Moreover, PCPs who served in township hospitals had significantly higher attitude scores than those who served in village clinics, which could be because PCPs, who served in township hospitals had greater accessibility to the latest medical knowledge.

We found some poor practices related to prediabetes among PCPs. In our survey, only 24.6% and 6.9% of the PCPs reported familiarity with the Chinese expert consensus, which recommends the interval for clinical follow-up and repeat laboratory assessments (17). Thus, very few PCPs were aware of the expert consensus, and addressing this problem may improve delivery of care for prediabetes. In a Diabetes Prevention Program study, lifestyle modification or metformin treatment was proven to be effective in reducing the incidence of diabetes and the risk of microvascular complications (9, 28). However, consistent with the results of previous studies (14–16), very few PCPs considered metformin use for prediabetes. This may be due to skepticism regarding the effectiveness of metformin (29).

The PCPs identified a lack of diet and exercise guidance as well as resource or financial limitations as important system-level barriers, and patient-related factors such as lack of recognition of prediabetes, uncertainty regarding the effectiveness of lifestyle modification, and lack of motivation as barriers to lifestyle modification. Therefore, goal-oriented measurements to tackle these barriers will be key to cost-effective management of prediabetes.

## Limitations

The present study had several limitations. First, this study was conducted among PCPs from the Central China region; due to variations in geographical and sociocultural characteristics across China, the results may not be generalizable. Second, the results of the KAP survey were self-reported by the participants, which may have introduced recall and social desirability biases, with more respondents reporting positive attitudes toward prediabetes.

## Conclusion

This study highlighted the substantial gaps in knowledge and practices regarding prediabetes among PCPs in the Central China region. The results also indicated underutilisation of prediabetes CME programmes by PCPs. Since PCPs play a crucial role in the prevention and management of diabetes, efforts to address these gaps in prediabetes knowledge and practice are of urgent importance. Structured programmes should be planned to improve prediabetes-related knowledge and practices among PCPs in China. In view of this, we make the following suggestions:

1. Educating providers on expert consensus regarding prediabetes, online education may be a candidate approach.
2. Develop a simplified prediabetes management algorithm by Chinese Diabetes Society.

## Data availability statement

The raw data supporting the conclusions of this article will be made available by the authors, without undue reservation.

## Ethics statement

The studies involving human participants were reviewed and approved by the Ethics Committee of the Second Xiangya Hospital of Central South. The patients/participants provided their written informed consent to participate in this study.

## Author contributions

XS and ZW designed the study. LP, DF, and YZ collected the data. LP conducted the data analysis and drafted the manuscript. XS, ZW, and ZZ revised the manuscript. JY carefully edited the revised manuscript. All authors read and approved the submitted version of the manuscript.

## References

[1] American Diabetes Association Professional Practice Committee and American Diabetes Association Professional Practice Committee. 2. Classification and diagnosis of diabetes: standards of medical care in diabetes-2022. Diabetes Care. (2022) 45(Suppl 1):S17–38. 10.2337/dc22-S002 34964875

[2] Li Y Teng D Shi X Qin G Qin Y Quan H. Prevalence of diabetes recorded in mainland China using 2018 diagnostic criteria from the American Diabetes Association: national cross sectional study. BMJ. (2020) 369:m997. 10.1136/bmj.m997 32345662 PMC7186854

[3] Tabák AG Herder C Rathmann W Brunner EJ Kivimäki M. Prediabetes: a high-risk state for diabetes development. Lancet. (2012) 379:2279–90. 10.1016/S0140-6736(12)60283-9 22683128 PMC3891203

[4] Gong Q Zhang P Wang J An Y Gregg EW Li H. Changes in mortality in people with IGT before and after the onset of diabetes during the 23-year follow-up of the da qing diabetes prevention study. Diabetes Care. (2016) 39:1550–5. 10.2337/dc16-0429 27411697 PMC5001147

[5] Rao Kondapally Seshasai S Kaptoge S Thompson A Di Angelantonio E Gao P Sarwar N. Diabetes mellitus, fasting glucose, and risk of cause-specific death. N Engl J Med. (2011) 364:829–41. 10.1056/NEJMoa1008862 21366474 PMC4109980

[6] Huang Y Cai X Mai W Li M Hu Y. Association between prediabetes and risk of cardiovascular disease and all-cause mortality: systematic review and meta-analysis. BMJ. (2016) 355:i5953. 10.1136/bmj.i5953 27881363 PMC5121106

[7] Echouffo-Tcheugui JB Narayan KM Weisman D Golden SH Jaar BG. Association between prediabetes and risk of chronic kidney disease: a systematic review and meta-analysis. Diabet Med. (2016) 33:1615–24. 10.1111/dme.13113 26997583

[8] Knowler WC Fowler SE Hamman RF Christophi CA Hoffman HJ Brenneman AT. 10-year follow-up of diabetes incidence and weight loss in the Diabetes Prevention Program Outcomes Study. Lancet. (2009) 374:1677–86. 10.1016/S0140-6736(09)61457-4 19878986 PMC3135022

[9] Knowler WC Barrett-Connor E Fowler SE Hamman RF Lachin JM Walker EA. Reduction in the incidence of type 2 diabetes with lifestyle intervention or metformin. N Engl J Med. (2002) 346:393–403. 10.1056/NEJMoa012512 11832527 PMC1370926

[10] Diabetes Prevention Program Research Group. Long-term effects of lifestyle intervention or metformin on diabetes development and microvascular complications over 15-year follow-up: the Diabetes Prevention Program Outcomes Study. Lancet Diabetes Endocrinol. (2015) 3:866–75. 10.1016/S2213-8587(15)00291-0 26377054 PMC4623946

[11] Centers for Disease Control and Prevention. National Diabetes Statistics Report, 2017. Atlanta, GA: Centers for Disease Control and Prevention, US Department of Health and Human Services (2017).

[12] Karve A Hayward RA. Prevalence, diagnosis, and treatment of impaired fasting glucose and impaired glucose tolerance in nondiabetic US adults. Diabetes Care. (2010) 33:2355–9. 10.2337/dc09-1957 20724649 PMC2963494

[13] Schmittdiel JA Adams SR Segal J Griffin MR Roumie CL Ohnsorg K. Novel use and utility of integrated electronic health records to assess rates of prediabetes recognition and treatment: brief report from an integrated electronic health records pilot study. Diabetes Care. (2014) 37:565–8. 10.2337/dc13-1223 24271190 PMC3898765

[14] Tseng E Greer RC O'Rourke P Yeh HC McGuire MM Albright AL. National survey of primary care physicians' knowledge, practices, and perceptions of prediabetes. J Gen Intern Med. (2019) 34:2475–81. 10.1007/s11606-019-05245-7 31502095 PMC6848700

[15] Tseng E Greer RC O'Rourke P Yeh HC McGuire MM Clark JM. Survey of primary care providers' knowledge of screening for, diagnosing and managing prediabetes. J Gen Intern Med. (2017) 32:1172–8. 10.1007/s11606-017-4103-1 28730532 PMC5653548

[16] Garay J Camacho PA Lopez-Lopez J Alvernia J Garcia M Cohen DD. Survey of knowledge for diagnosing and managing prediabetes in Latin-America: cross-sectional study. Diabetol Metab Syndr. (2019) 11:102. 10.1186/s13098-019-0500-4 31827627 PMC6894241

[17] Chinese Chinese Society of Endocrinology Chinese Diabetes Society Chinese Endocrinologist Association Endocrine Endocrine and Metabolic Disease Branch of Chinese Research Hospital Association Diabetes Diabetes Branch of Chinese Research Hospital Association. Intervention for adults with pre-diabetes: a Chinese expert consensus. Chin J Endocrinol. (2020) 36:371–80. 10.3760/cma.j.cn311282-20200115-00022

[18] National Health and Family Planning Commission. Notice of the National Health and Family Planning Commission on printing and distributing the national basic public health service standard (Third Edition) China. Beijing: National Health and Family Planning Commission, PRC (2017).

[19] Maruthur NM Ma Y Delahanty LM Nelson JA Aroda V White NH. Early response to preventive strategies in the Diabetes Prevention Program. J Gen Intern Med. (2013) 28:1629–36. 10.1007/s11606-013-2548-4 23860722 PMC3832727

[20] Hamman RF Wing RR Edelstein SL Lachin JM Bray GA Delahanty L. Effect of weight loss with lifestyle intervention on risk of diabetes. Diabetes Care. (2006) 29:2102–7. 10.2337/dc06-0560 16936160 PMC1762038

[21] PCC Care. Prevention or delay of type 2 diabetes and associated comorbidities: standards of medical care in diabetes-2022. Diabetes Care. (2022) 45(Suppl 1):S39–S45. 10.2337/dc22-S003 34964876

[22] Niroomand M Ghasemi SN Karimi-Sari H Khosravi MH. Knowledge, attitude, and practice of Iranian internists regarding diabetes: a cross sectional study. Diabetes Metab J. (2017) 41:179–86. 10.4093/dmj.2017.41.3.179 28657233 PMC5489498

[23] Khan AR Al Abdul Lateef ZN Khamseen MB Al Aithan MA Khan SA Al Ibrahim I. Knowledge, attitude and practice of ministry of health primary health care physicians in the management of type 2 diabetes mellitus: a cross-sectional study in the Al Hasa District of Saudi Arabia, 2010. Niger J Clin Pract. (2011) 14:52–9. 10.4103/1119-3077.79241 21493993

[24] Aghili R Malek M Baradaran HR Peyvandi AA Ebrahim Valojerdi A Khamseh ME. General practitioners' knowledge and clinical practice in management of people with type 2 diabetes in Iran; the impact of continuous medical education programs. Arch Iran Med. (2015) 18:582–5. 26317599

[25] Alduraibi RK Almigbal TH Alrasheed AA Batais MA. Knowledge, attitudes, and practices of primary health care physicians regarding the pre-travel counselling of patients with type 2 diabetes in Riyadh, Saudi Arabia. BMC Fam Pract. (2020) 21:200. 10.1186/s12875-020-01273-z 32972370 PMC7517797

[26] Mahmoud K Jaramillo C Barteit S. Telemedicine in low- and middle-income countries during the COVID-19 pandemic: a scoping review. Front Public Health. (2022) 10:914423. 10.3389/fpubh.2022.914423 35812479 PMC9257012

[27] Jafarzadeh-Esfehani R Mirzaei Fard M Habibi Hatam-Ghale F Rezaei Kalat A Fathi A Shariati M. Telemedicine and computer-based technologies during coronavirus disease 2019 infection; a chance to educate and diagnose. Arch Iran Med. (2020) 23:561–3. 10.34172/aim.2020.60 32894969

[28] Diabetes Prevention Program Research Group. Long-term safety, tolerability, and weight loss associated with metformin in the Diabetes Prevention Program Outcomes Study. Diabetes Care. (2012) 35:731–7. 10.2337/dc11-1299 22442396 PMC3308305

[29] Mainous AG 3rd Tanner RJ Scuderi CB Porter M Carek PJ. Prediabetes screening and treatment in diabetes prevention: the impact of physician attitudes. J Am Board Fam Med. (2016) 29:663–71. 10.3122/jabfm.2016.06.160138 28076248

