# Supplementary material for: Primary care providers' knowledge, attitudes, and practices related to prediabetes in China: A cross-sectional study
Source: Front Public Health. 2023 Feb 23;11:1086147. doi: 10.3389/fpubh.2023.1086147 (PMC9995854; doi:10.3389/fpubh.2023.1086147)
Supplement: Supplementary file 1 [file Table_1.DOCX]

**Survey of Primary Care Providers’ knowledge, attitudes, and practices related to prediabetes**

We would invite you to participate a survey conducted by the Second Xiangya Hospital of Central South University. This study is part of an effort to evaluate primary care providers’ knowledge, attitudes, and practices related to prediabetes.

This survey is confidential and your individual responses will not be identifiable. Your information will only be seen by researchers. Completion of this survey is entirely voluntary and will serve as your consent to take part in this research study. This study was approved by the Ethics Committee of the Second Xiangya Hospital of Central South University.

Thank you very much for your participation in this important study!

**Part I. Demographic information**

Age: years Gender: □ male □ female

Practice setting: □ Township hospital □ Village clinic

Professional titles: □ Resident physicians □ Attending physicians □ Senior physicians

Duration of practice: years

CME attendance during the past year regarding diabetes: □ yes □ no

Positive family history of diabetes: □ yes □ no

**Part II. The following questions apply to your knowledge regarding prediabetes**

1. Is prediabetes an intermediate stage between normal glycemia and diabetes?

□ Correct □ False □ Not sure

1. Which one is not a risk factor in prediabetes screening?

□ Sedentary lifestyle

□ family history of diabetes in a first-degree relative

□ Sedentary lifestyle

□ Obesity/ overweight

□ Hyperuricemia

1. Which one is the prediabetes laboratory criterion for fasting glucose level?

□ 6.1mmol/L≤FBS<7.0mmol/L

□ FBS<6.1mmol/L

□ 7.0mmol/L≤FBS<12.0mmol/L

□ 12.0mmol/L≤FBS

1. Which one is the prediabetes laboratory criterion for the 2-h PG level during OGTT?

□ 6.1mmol/L≤2-h PG <7.8mmol/L

□ 7.8mmol/L≤2-h PG <11.1mmol/L

□ 11.1mmol/L≤2-h PG <15.0mmol/L

□ 15.0mmol/L≤2-h PG

1. Which one is the prediabetes laboratory criterion for HbA1c level?

□ 5.7%-6.4%

□ 6.1%-7.0%

□ ＜6.5%

□ ＜7.0%

1. Which one is the correct body weight loss recommendation for individuals with prediabetes during 3-6months?

□ 3-5%

□ 5-7%

□ 10-15%

□ >15%

**Part III. The following questions apply to your attitudes regarding prediabetes**

1. Prediabetes is associated with a high risk of progression to overt type 2 diabetes

□ Strongly disagree

□ Disagree

□ Neutral

□ Strongly agree

□ Agree

1. Prediabetes is associated with an increased risk of premature mortality

□ Strongly disagree

□ Disagree

□ Neutral

□ Strongly agree

□ Agree

1. Most individuals with prediabetes have not been diagnosed

□ Strongly disagree

□ Disagree

□ Neutral

□ Strongly agree

□ Agree

1. Prediabetes is reversible

□ Strongly disagree

□ Disagree

□ Neutral

□ Strongly agree

□ Agree

1. Regular exercise helps delay or prevent the transition from prediabetes to diabetes

□ Strongly disagree

□ Disagree

□ Neutral

□ Strongly agree

□ Agree

1. Metformin helps delay or prevent the transition from prediabetes to diabetes

□ Strongly disagree

□ Disagree

□ Neutral

□ Strongly agree

□ Agree

**Part IV. The following questions apply to your practices regarding prediabetes**

1. Which one is your initial suggestion for individuals with prediabetes?

□ Provide counseling on diet changes and physical activity to lose weight

□ Discuss with patient about treatment with metformin

□ Refer the patient to bariatric surgery

□ Refer the patient to a behavioral weight loss program

1. How long do you recommend individuals with prediabetes to repeat laboratory work?

□ Three months

□ Six months

□ One year

□ Two years

1. How long do you recommend individuals with prediabetes to return for follow-up clinic visit?

□ Three months

□ Six months

□ One year

□ Two years

1. Which one is your suggestion for a patient who fail to respond to lifestyle modification?

□ Discuss with patient about treatment with metformin

□ Refer the patient to bariatric surgery

□ Refer the patient to a behavioral weight loss program

□ Others

**Part V. The following questions apply to barriers to lifestyle modification**

Select the options you think are impediments to lifestyle change and list them if there are others.

- Lack of recognition of the harm posed by prediabetes
- Lack of diet and exercise guidance
- Uncertainty of the effectiveness of lifestyle modifications
- lack of motivation

If there are other possible potential barriers you encountered in daily clinical practice， please list them in the following
